# The Bar of History: "The Hospital's" Review of 1912

**Published:** 1913-01-04

**Authors:** 


### THE BAR OF HISTORY.

### "The Hospital's" Review of 1912.

It is rather because so much is recorded than that so much has been achieved which makes the task of reviewing the work and developments in hospitals and medical matters during any past year at first sight seem a little overwhelming. Before the bar of history much of this will pale, but our task is to summarise what took place rather than to pass judgment upon it.

### Vivisection and Secret Remedies.

In one respect the close of 1911 and the close of 1912 are similar; both were signalised by stormy meetings of the constituents of the Metropolitan Hospital Sunday Fund, and in both cases the rigidity of the constitution of the Fund was the bone of contention. Last year the constitution was "called over the coals" in relation to the attitude of the Fund to St. George's Hospital, and last week Mr. Lewis's resolution dealing with the alleged destruction of the subscribers' control and of all true representation provoked the outburst. Again, the position into which Guy's Hospital has been forced by the tactics and attitude of the Southwark Board of Guardians is as fruitful of bitterness, misrepresentation, and confusion in the public mind as it was last January. During the year a Royal Commission and a Select Committee dealing with highly contentious questions have respectively published and taken evidence for their Reports. The issue of the Report of the Royal Commission on Vivisection, however much either party may claim its sanction, has been followed by no diminution of rancour, and in fact both sides have declared that their point of view has been vindicated. Matters are really very much where they were on this question. The second Commission, which has been engaging the attention of the hospital and medical worlds, the Select Committee on Secret Remedies, is still receiving evidence, and its Report may not be expected for several months. The chief value of the Commission up to date may be said to have been the emphasis and publicity which it has given to facts which were previously well known to experts but which are now common lay property. The extent of the traffic in proprietary medicines has been established with a startling array of figures, and the best means of protecting the public from its own greedy credulity have been discussed from every point of view. The lay press, for obvious reasons, has tended rather to veil the proceedings by a conspiracy of silence, but the force of truth is great, and what has leaked through has been readily assimilated.

### Sanatoria.

The various anti-phthisis campaigns in Edinburgh, Wales, Bradford, and in a minor degree in every county, have continued to obtain great publicity and to attract very large financial support from the public. The movement in favour of school clinics has grown steadily, and Cardiff and Portsmouth have been the provincial centres which, perhaps, have given birth to the most far-reaching proposals on this head. The rival camps in favour of sanatorium treatment and of tuberculin have been busy attacking each other, but the need for institutional treatment under the Insurance Act has led to the conversion of several unoccupied isolation hospitals to sanatoria purposes, while a network of tuberculous dispensaries is being laid in almost every city and urban area. It is in these dispensaries that the tuberculin advocates are looking for their opportunity. The question of shelters for open-air patients has also led to the establishment of an important institutional truth, that, namely, the shelter made on the premises by the sanatorium or handy-man usually proves cheaper and more suitable than any commercial variety because the needs of any two sanatoria are hardly ever the same.

### New Institutions of the Year.

Among the mass of building which has been undertaken during 1912 one or two of the chief institutions alone can be mentioned. Probably the largest provincial hospital to be opened during the year was the King Edward Memorial Infirmary at Bristol, which was described in detail in The Hospital last July. It claims to be the largest surgical unit in Europe and virtually replaces the traditional Royal Infirmary, of which it was at first described as an extension. Mr. Percy Adams and Mr. Charles Holden were its architects. The most unique new institution of the year was, however, the Research Hospital which was opened at Cambridge in May, largely thanks to Mr. R. C. Brown, F.R.C.P., F.R.C.S., Mr. T. S. P. Strangeways, and Miss Sykes, of Huddersfield, for the prosecution of research by modern laboratory and clinical methods in rheumatoid arthritis and the diseases allied to it, which neither the hospitals in general nor the physician in private practice have been able for various reasons to undertake in a satisfactory way. The patients are also unique, as they enter the hospital knowing that the mysterious nature of their disease demands experiment, and that the object of their stay is to provide clinical material for the research workers. Among important institutions decided on or actually being built the new Maudsley Mental Hospital at Denmark Hill demands special mention. Here, too, one of the main objects is research work. The London County Council, moreover, is building a new institution on the Horton Estate, and London itself gained impressiveness at the corner of Wimpole Street when the new premises of the Royal Society of Medicine were opened there last May in the presence of the King. The London Hospital's new Preliminary Training School at Tredegar House, Bow, may also be mentioned. Among the many small institutions which have been opened or suggested we can note only two. The proposed new South London

Hospital for Women, which is to be built at Clapham, and is intended to do for South London's women patients what the New Hospital for Women in Euston Road does for the North; and the St. Andrew's Hospital which a Roman Catholic benefactress has empowered Cardinal Bourne to open. The year 1912, however, has been a year of hospital migration, so far as London is concerned at any rate. The new King's College Hospital at Denmark Hill is nearing completion steadily; Westminster Hospital has published its decision to "go," and Broad Sanctuary will never have a hospital overlooking it again probably; and, lastly, the British Lying-in Hospital in Endell Street has decided to amalgamate with the Home for Mothers and Babies at Woolwich.

### General Events.

The principal lay happenings, whose echoes have reverberated in the hospital and medical worlds, were undoubtedly the *Titanic* disaster, by which several medical men lost their lives, and the "hunger-strikes" of imprisoned women suffragists which brought the medical methods of forcible feeding into prominence and criticism. The public, of course, could not realise that such methods are of constant employment in, for example, certain cases of mental disease, but the protagonist in the controversy which really deserved sympathy was not so much the doctor who was called to aid the State in the matter, nor even, profoundly considered, the patient herself, but the Cabinet which sanctioned an unpopular form of coercion because it feared a popular storm of protest were its women prisoners allowed to persist in their refusal to take food until death supervened. The Cabinet was not strong enough to face the charge of permitting suicide, and thought, perhaps wisely, that the charge of active cruelty would create less odium in the popular mind. In the end the prisoners had only to show signs of illness as a result of voluntary starvation followed by forcible feeding to give the Cabinet the excuse for which it was waiting—namely, to let them out. The two chief strikes during the year, the coal strike and the London Dock strike, fortunately did not affect the hospitals so much as had been feared. In the coal strike hospitals had either accumulated sufficient supplies to tide them over, or special gifts, in some cases special permission to receive coal, were accorded to them. In the Dock strike, however, one London institution, the Poplar Hospital for Accidents, suffered severely through the stopping of its hydraulic lift owing to sudden cessation of work at the Port of London Authority's power-station. Three patients were actually in the lift at the moment when the power was cut off, and apart from the difficulty of getting these to their wards, the hospital's work was disorganised, as its stairs are narrow and no other means besides the derelict lift but carrying by hand were available. Special porters had to be engaged. It is to be added, moreover, that the public at large was not affected by the strikes to anything like the extent which was first thought probable. The general medical question most eagerly seized on by the lay newspapers was Sir Almroth Wright's personal opinions of feminine psychology, to which the militant methods of the women suffragists at the time he undertook to write gave a factitious prominence. Another question raised once or twice, and which is likely to be heard discussed in lay circles before very long, is the question, "What is an unjustifiable operation?" The practical impossibility of finding any definition sufficiently definite to be workable and sufficiently loose to allay opposition gives to this question a pleasant haziness likely to commend it to newspaper editors and readers when a restful substitute for thought is required as August again comes round.

### Parliament.

Legislation as regards both hospitals and the medical profession has come to be narrowed down in the past twelve months to mean nothing but the Insurance Act. Of all that has been said and done concerning it we will only recall this. It became an Act on July 15, 1912. It excited the bitter hostility of the profession, which has forced some concessions from the Chancellor of the Exchequer. It has cost the voluntary hospitals many subscriptions, and to-day, a fortnight before it is nominally to come into operation so far as the bestowal of "benefits" are concerned, the voluntary hospitals have not collectively decided what attitude towards insured patients they will adopt. The various steps which have led to this result would take too long to record. Are they not to be found in the weekly issues of The Hospital itself? Apart from the Insurance Act, however, workmen's compensation cases have been of very frequent occurrence, and the importance of the medical witness has grown in consequence.

### Mental and Fever and Poor Law.

In the mental hospital world the claim for research laboratories has grown insistent, and a scheme has been propounded for linking up the provincial mental hospitals in this respect. The claim, too, for a sixty-hour week has grown in volume, for the medical superintendents most suspicious of legal interference with the very delicate adjustment necessary in the work of their institutions have come to see that an eighty-four hour week, which still exists in some institutions, must be mended by legislation if it cannot be mended in any other way. The strain of mental work on women is at any time very great. The movement for one day's rest in seven has gained another victory at Cardiff Mental Hospital, where, too, certain moneys in lieu of rations have been granted to married workers for each day's leave. Parliament has closed the year by tumbling a stone of disturbance into the waters of mental-hospital content in the shape of a Feeble-minded Bill which, as at present drawn, satisfies very few superintendents, and with the possibilities that it holds out for the large increase of institutional accommodation is likely to prove staggering in cost, should it ever become law more or less in its present form.

The fever hospital has been devoting its attention to the best means of reducing the cross-infection which at present shows rather badly in the statistics of certain institutions, and here, too, the demand for more research work into the causes of the diseases in its province is growing in insistency. It is felt that the fever hospital must be more than an isolation hospital, and must undertake more actively the study of preventive methods. To the Poor-Law infirmary the end of the year has brought also a surprise in the form of the Draft Poor-Law Order which, if it receives official sanction, will do something for the autocracy of the medical officer and the superintendent nurse in the sick wards of unseparated infirmaries. It is, however, only a half-way house to the complete separation of the infirmary from the workhouse proper and depends too much upon tact and co-operation, which muddled functions of medical and administrative staffs have rendered often so far to seek in the past, to prove a permanent solution of the difficulties. If sanctioned, however, it will do something to remove existing causes of friction; the day of complete separation, which is inevitable, has yet to come. The status and pay of Poor-Law dispensers has improved during the year; they have won recognition of their qualification by the title pharmacist in several more institutions. Their pay in many cases has also been increased. Important new infirmaries are springing up at several new centres, among which the Bristol scheme for the classification of Poor-Law patients in separate institutions, and the recent effects of enlarging the boundaries of the City of Birmingham in the same direction must be specially recorded.

### The Out-patients' Question.

The long expected Report on the Reform of Out-patients, which was undertaken by a Special Committee appointed by King Edward's Hospital Fund for London, appeared during the latter part of the year. Its general effect was to approve the almoner system and the reform of out-patient departments on consultative lines, and to advocate the co-operation of the hospital almoner with every existing accredited agency for the after-care or economic rehabilitation of the patient at the time of his or her discharge. In September the third annual Conference of the British Hospitals Association was held at Birmingham and papers were read on "Hospitals and the State" (by Sir William Collins), "Hospital Management in Relation to Responsibility" (by Mr. Power), "Hospitals and the Insurance Act" (by Dr. Nathan Raw, of Liverpool, and Dr. Basil Rhodes, of the South Staffordshire Infirmary), and on "The Almoner" (by Mr. E. S. Kemp). The Conference was a great success, and, if one glance ahead be permitted to this page of memory, the next and fourth will take place at Oxford.

Turning now to more personal matters the year has seen an accession of medical men to the ranks of political candidates, and these, it is interesting to record, have shown a preference for the Labour or Socialist programmes. The profession, indeed, has taken a much more prominent part in public life, as though its experiences with the Chancellor over the Insurance Act had proved a fretful teething period which were rapidly leading to its contented mastication of public affairs. Thus 1912 saw the first medical man in the office of Lord Mayor of London, and Sir Thomas Boor Crosby proved how useful to the hospital world a medical Lord Mayor could be. Dr. Morrison again has represented his profession in the highest circles of international State-craft, having become the political adviser to the President of the Chinese Republic. A Life of Florence Nightingale, we also note, has been promised, Sir E. T. Cooke acting as biographer.

### The Year's Death-roll.

On the other hand, the death-roll has been heavy. Lister has died full of years and honours, the first medical man, by the way, to win a peerage as a surgeon. Sir Henry Butlin, P.R.C.S.; Sir Willian Allchin, the well-known examiner; Dr. Sophia Jex-Blake; Sir F. C. Wallis, of general surgery an Union Jack Club fame; Sir William Thornley, well known in Ireland; Sir William Murrell, the author of the famous "What to do in Cases of Poisoning"; Dr. Huggard, of Davos; Dr. James Scott Sequeira, a hero of the cholera epidemic; Mr. Clinton Dent, a veteran of St. George's Hospital; Mr. John Taylor, a veteran surgeon of Chester Infirmary; Mr. Troutbeck, the famous non-medical coroner: these names at once make a noteworthy obituary. But there are others: Dr. J. Dixon Mann, Professor of Forensic Medicine at Victoria University, Manchester; Dr. William Ogle, who left Holy Orders for medicine and medicine for the Registrar-General's Office; Mr. L. A. Bidwell, the great post-graduate teacher; and Dr. R. E. Thompson, known to London and Canada in the widely different spheres of veteran physician and pioneer of the Canadian West. These are dead also and excite the hope that their successors may have equally variegated and satisfying records when they, too, fall victims to the obituary writer, the only correspondent of the lay press who never seems to die.

### Secretaries who have Died.

Hospital secretaries also have lost more than one noteworthy personality. Mr. Arthur Edward Reade, a former secretary of Charing Cross Hospital and a well-known friend of the older generation of secretaries in London; Mr. Samuel Welsh, founder and former secretary of Walsall and District Hospital; and, lastly, Mr. J. Francis Pink, late secretary of the Royal Dental Hospital, Leicester Square, and formerly secretary and librarian of Charing Cross Hospital Medical School: all these have died—Mr. Pink only in the middle of December—and remind us of the valuable work that is quietly done. Let "life levels all men; death reveals the eminent" be their epitaph. It is the only motto we know which makes an equally cheering appeal to the dead and to the living.

